# Adult Male with Neck Pain

**DOI:** 10.5811/cpcem.2017.3.33250

**Published:** 2017-07-14

**Authors:** Shaw Natsui, Sarah E. Frasure

**Affiliations:** Brigham and Women’s Hospital, Department of Emergency Medicine, Boston, Massachusetts

## CASE PRESENTATION

**Image 1:**
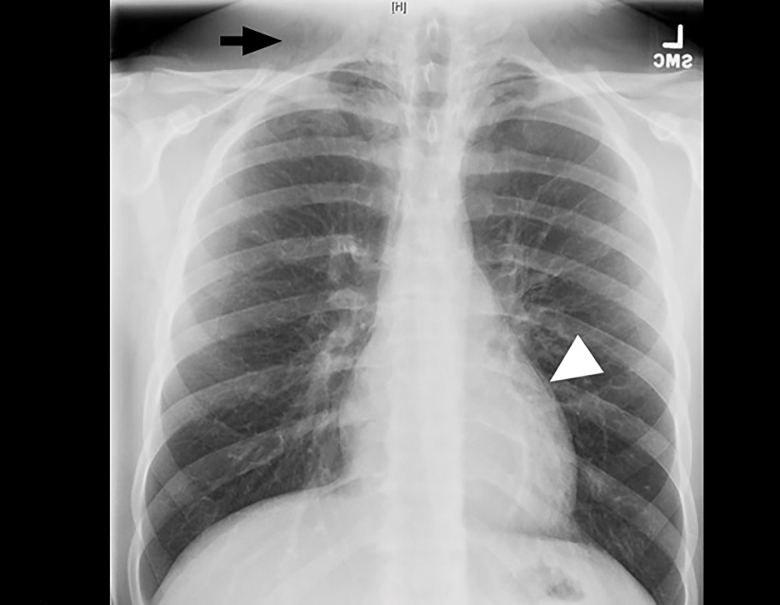
Plain radiograph demonstrates subcutaneous emphysema (arrow) and pneumomediastinum (arrowhead).

**Image 2:**
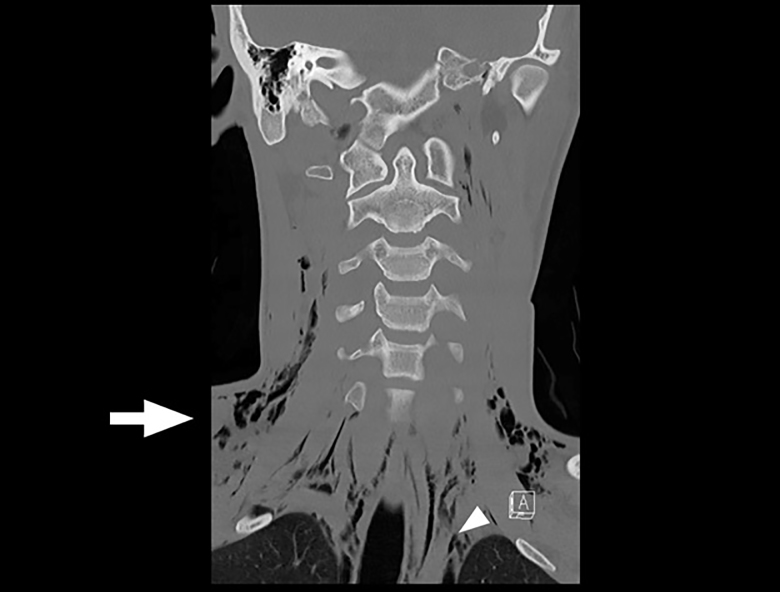
Coronal view of computed tomography of the patient’s neck demonstrates subcutaneous emphysema (arrow) and pneumomediastinum (arrowhead).

**Image 3:**
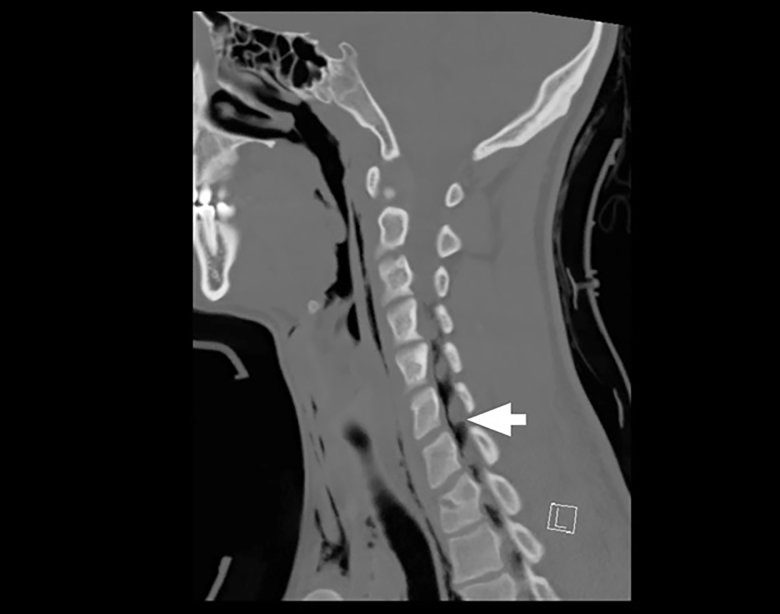
Sagittal view of the patient’s neck computed tomography demonstrates pneumorrhachis (arrow).

A 19-year-old male presented to the emergency department after a fall while playing soccer. He attempted to head the ball but instead fell backwards, hitting his head and neck on the ground. He did not lose consciousness but developed immediate pain along the right side of his neck. He also described pleuritic chest pain. He denied vision changes, nausea, vomiting, dyspnea, abdominal pain, paresthesias, or extremity weakness. His vital signs were normal. His physical exam was notable for palpable crepitus over the right supraclavicular area. He had clear breath sounds bilaterally. His neurologic exam was intact. Laboratory tests were unremarkable. A chest radiograph was performed (Image 1), and the patient was subsequently evaluated by the trauma service. Thereafter, a computed tomography of the neck, chest, abdomen, and pelvis was performed (Images 2 and 3), and the patient was admitted to the hospital.

## DISCUSSION

*Pneumomediastinum and pneumorrhachis*. The patient was admitted to the surgical service for close airway monitoring and a barium-swallow study to assess for esophageal perforation. No surgical intervention was necessary. Pneumorrhachis is the presence of air within the spinal canal and was first described in 1977.^1^,^2^ Conditions that produce elevated intrathoracic pressure, such as coughing, sneezing or vomiting, can lead to the entrapment of air in the paraspinal soft tissues and epidural space via the neural foramina.^3^ The majority of cases appear to involve young males.^1^,^4^ The association between pneumorrhachis and subcutaneous emphysema due to barotrauma is well described. The mechanism behind the association of pneumomediastinum with pneumorrhachis, however, is poorly understood.^1^ Related phenomena include pneumocephalus, pneumothorax, and pneumopericardium. Given the rarity of pneumorrhachis no standard management guidelines exist.^5^ Fortunately, the majority of cases of pneumorrhachis are self-limiting and successfully managed without surgical intervention.

CPC-EM Capsule

What do we already know about this clinical entity?

Pneumorrhachis is the presence of air within the spinal canal, caused by conditions that create elevated intrathoracic pressure.

What is the major impact of the image(s)?

The images illustrate the association of pneumorrhachis with other pathology such as subcutaneous emphysema and pneumomediastinum.

How might this improve emergency medicine practice?

The presence of subcutaneous emphysema or pneumomediastinum on plain film imaging for a trauma patient should raise suspicion for associated pneumorrhachis.
